# Essentiality of Early Diagnosis of Molar Incisor Hypomineralization in Children and Review of its Clinical Presentation, Etiology and Management

**DOI:** 10.5005/jp-journals-10005-1164

**Published:** 2012-12-05

**Authors:** Nishita Garg, Abhay Kumar Jain, Sonali Saha, Jaspal Singh

**Affiliations:** Lecturer, Department of Pedodontics, Sardar Patel Postgraduate Institute of Dental and Medical Sciences, Lucknow, Uttar Pradesh India, e-mail: drnishita_garg@yahoo.com; Lecturer, Department of Orthodontics, Sardar Patel Postgraduate Institute of Dental and Medical Sciences, Lucknow, Uttar Pradesh, India; Lecturer, Department of Pedodontics, Sardar Patel Postgraduate Institute of Dental and Medical Sciences, Lucknow, Uttar Pradesh, India; Reader, Department of Pedodontics, Teerthanker Mahaveer Dental College and Research Centre, Moradabad, Uttar Pradesh, India

**Keywords:** Hypomineralization, First permanent molars, Post-eruption breakdown

## Abstract

Molar incisor hypomineralization (MIH) is a common developmental condition resulting in enamel defects in first permanent molars and permanent incisors. It presents at eruption of these teeth. One to four molars, and often also the incisors, could be affected. Since first recognized, the condition has been puzzling and interpreted as a distinct phenomenon unlike other enamel disturbances. Early diagnosis is essential since, rapid breakdown of tooth structure may occur, giving rise to acute symptoms and complicated treatment. The purpose of this article is to review MIH and illustrate its diagnosis and clinical management in young children.

**How to cite this article:** Garg N, Jain AK, Saha S, Singh J. Essentiality of Early Diagnosis of Molar Incisor Hypomineralization in Children and Review of its Clinical Presentation, Etiology and Management. Int J Clin Pediatr Dent 2012;5(3):190-196.

## INTRODUCTION

Enamel defects are known to occur due to depressed activity of the enamel-forming ameloblasts which result in the formation of linearly distributed pits or grooves. These alterations can be found in two different stages: Enamel matrix formation (secretion phase) and enamel mineralization (maturation phase). If an unbalance occurs during the secretion phase, the enamel defect is called hypoplasia. If it occurs during the maturation phase, it is called hypomineralization. Once formed, enamel is not remodeled during life and every individual’s enamel is a record of the first 8 or 9 years of their life when the crowns are formed.^1^ In hypoplasia only the tooth surface is involved that can be considered as an external defect associated to the smallest thickness of the affected enamel. It can be present as shallow or deep fossae with horizontal or vertical grooves and with partial or total absence of enamel. Hypomineralization on the other hand presents as an anomaly in the tissue translucency. A white or yellowish/brownish area can be seen and there is no thickness alteration. Recently one enamel alteration of great clinical significance affecting the first permanent molars (FPM) was described in four presentations at the European Academy of Pediatric Dentistry Congress in 2000. These reports called the condition hypomineralized FPM,^2^ idiopathic enamel hypomineralization in FPM,^3^ nonfluoride hypomineralization in FPM^4^ and cheese molars.^5^ It was defined as a single clinical entity and termed molar incisor hypomineralization (MIH). It is defined as hypomineralization of systemic origin affecting one, two, three or all first permanent molars and the permanent incisors.^6^ Due to the lack of an agreed definition, prior to 2001, the literature regarding MIH is confusing and it is difficult to be sure that different researchers are referring to the same thing. The severity of MIH may vary greatly. It ranges from mild opacities to posteruptive breakdown. It may be asymmetrical but should an FPM be severely affected the contralateral molar is more likely to be affected. In affected incisors, the severity of hypomineralization is usually less than that of the affected molars.^5^ Literature is scanty on this highly variable ectodermal disorder, supra added by its manifold clinical features this leads to many undiagnosed cases which lead to an array of clinical errors. Solving this problem and possible consequences can be a great challenge for professionals as treatment can be complex. The purpose of this review is to describe the diagnosis, clinical features, prevalence, putative etiological factors of MIH and to describe sequential treatment options all of which may lead to existing literature on the topic and update pedodontists role in management of such clinical disorders.

## MIH DIAGNOSIS

Any examination for MIH should be undertaken on clean wet teeth and the age of 8 years is optimum, as at this age all permanent first molars and most of the incisors are erupted. In addition, the permanent first molar teeth will be in a relatively good condition without excessive posteruptive breakdown. Judgments related to individual teeth (all FPM and incisors) should be recorded, helping in the correct diagnosis of the condition.^6^ Diagnostic criteria for hypomineralization of FPMs currently available are the modified defect of dental enamel (DDE) index given by federation dentaire international (Table 1) and the criteria of Weerheijm et al^6^ (Table 2).

## DIFFERENTIAL DIAGNOSIS

Teeth with developmental defects of enamel may present similarly, regardless of etiology, and the development defects of enamel hypoplasia may be confused with MIH. Enamel hypoplasia is a quantitative defect associated with reduced localized thickness of enamel whereas hypomineralization is a qualitative defect affecting enamel translucency.^7^ Diagnostically, MIH and enamel hypomineralization (EH) can be difficult to differentiate when affected molars have posteruptive enamal breakdown (PEB) due to caries or masticatory trauma. In a child with high caries rate, MIH can be masked by extensive caries or restorations. PEB may lead to a clinical picture resembling hypoplasia. However, in hypoplasia, the borders of the deficient enamel are smooth, while in posteruptive enamel breakdown the borders to normal enamel are irregular.^6,8^ MIH can also be confused with fluorosis, however, the enamel opacities of fluorosis are diffuse, in contrast to the well-demarcated borders of hypomineralized enamel seen in MIH.^6^ In addition, fluorosed enamel is caries resistant; in comparison to the caries prone MIH-affected enamel. Furthermore, the difference between MIH and amelogenesis imperfect (AI) is one of definition. In AI, all teeth are affected and may be detected preeruptively on radiograph. Generally, affected FPM’s with MIH are asymmetrical. There is usually a positive family history in cases of AI.^6,8^

**Table 1:** **Table 1:** Modified DDE index (FDI 1992)

Mild		<30% of the tooth’s enamel surface area visibly disrupted (this encompasses the entire range reported in most other studies)	
Moderate		31 to 49% of the tooth’s enamel surface area visibly disrupted	
Severe		>50% of the tooth’s enamel surface area visibly disrupted	

**Table 2:** **Table 2:** Definitions of the criteria used for diagnosing MIH (Weerheijm et al 2001a)

*Criteria*		*Definitions*	
Opacity		A defect involving an alteration in the translucency of the enamel, variable in degree. The defective enamel is of normal thickness with a smooth surface and can be white, yellow or brown in color. The border of the lesions is demarcated.	
PEB		A defect that indicated deficiency of the surface after eruption of the tooth. This may be caused by such factors as trauma and attrition.	
Atypical restoration		Size and shape of restoration do not conform to typical restorative characteristics. In most cases, restorations will be extended to the buccal or the palatinal smooth surface. At the border of the restoration, opacity may be noticed.	
Extraction due to MIH		Absence of a molar should be related to the other teeth of the dentition. Absence of a first permanent molar in a sound dentition is suspected to have been an MIH molar.	

## CLINICAL APPEARANCE, SYMPTOMS AND SIGNS ASSOCIATED WITH MIH

Clinical appearance, symptoms and signs associated with MIH:

- Large demarcated opacities, whitish-cream or yellow- brown in color
- May or may not be associated with posteruption enamel breakdown
- Hypersensitivity
- Difficult to anesthetize
- Rapid caries progression.

Clinically, the lesions of MIH are fairly large demarcated opacities of altered enamel translucency. The defective enamel is white-cream or yellow-brown in color. The opacities are usually limited to the incisal or cuspal one third of the crown, rarely involving the cervical one third. The intact enamel surface is typically hard, smooth and often hypermineralized following posteruptive maturation; the subsurface enamel is soft and porous.^6,7^ The MIH-affected FPM’s are sometimes hypersensitive to stimuli and may be difficult to anesthetize. It is believed that there is subclinical pulpal inflammation due to porosity of the enamel which could lead to hypersensitivity experienced by som individuals.^6,9^ In a study comparing the pulps of noncarious hypomineralized FPM to apparently sound FPM from MIH affected individuals, it was concluded that the changes were indicative of inflammatory changes.^10^ Caries progression is very rapid in FPM affected by MIH due to the porous enamel. It is further compounded by the fact that these affected children may avoid brushing because of sensitivity.^2,6^

## PUTATIVE ETIOLOGICAL FACTORS

Majority of previous studies imply that the etiology of MIH is complex with undetermined systemic and genetic factors disrupting normal amelogenesis in the affected teeth. A variety of systematically acting medical factors proposed as contributing to or causing MIH includes prenatal, perinatal and postnatal illnesses, low birth weight, antibiotic consumption and toxins from breastfeeding. In an attempt to explain the possible etiological factors it is important to remember that between 28 weeks *in utero* and the first 10 days of life ameloblasts initiate amelogenesis in the first permanent teeth to be formed, the FPM, followed by the other teeth later in time. If the function of ameloblasts is interrupted, temporarily or permanently, then depending upon the time of insult, enamel hypoplasia or enamel hypomineralization is produced. Experiments have shown that conditions affecting the enamel matrix pH, i.e. respiratory acidosis and abnormal oxygen levels resulting from hypoventilation in various respiratory diseases inhibit the action of the proteolytic enzymes and the development of the crystal hydroxyapatite resulting to enamel hypomineralization. Lack of calcium phosphate in the area of the crystallites might result in reduced calcium deposits and lower ratio of calcium/phosphorus leading again to enamel hypomineralization. Maternal pyrexia has been shown experimentally to have a detrimental influence on amelogenesis, ranging from ameloblastic dysfunction to complete cellular degeneration.^11^ In case of maternal diabetes hypocalcemia in the mother and oxygen shortage problems to the infant may result in enamel hypomineralization.^12^ Prenatal conditions such as prolonged maternal nausea and vomiting jeopardize fluids and electrolytes, as well as nutritional status leading occasionally to fetal biochemical disturbance. Use of myometrium spasmolytics medication may produce side-effects, such as nausea, vomiting and fetal hypocalcemia which may again disrupt amelogenesis.^13^ Perinatal medical conditions appear to be associated with hypocalcemia and hypoxia. Previous studies have shown that early neonatal hypocalcemia is present in approximately 30 to 75% of cases of preterm low birth neonates, particularly in those with respiratory distress and birth asphyxia due to complicated, prolonged or difficult delivery.^14,15^ The reason is that two-thirds of an individual’s stores of calcium and phosphorus accumulate during the last trimester of pregnancy and preterm infants miss much of this mineral accretion.^16^ In a previous study it was shown that compared with newborns delivered vaginally, those delivered by elective cesarean section, at around full-term had an increased risk of overall and serious respiratory illnesses, conditions often associated with hypoxia.^17^ Also, the commonly used spinal anesthesia for cesarean section has a frequent complication of maternal hypotension that can be associated with severe nausea or vomiting which occasionally produces infant hypoxia.^14^ Postnatal special attention has been paid to infectious childhood illnesses, high fever, medication (antibiotics), environmental toxicants, breastfeeding and use of fluorides. Illnesses such as otitis media,^18^ pneumonia^18,19^ asthma,^19^ urinary tract infections and chicken pox which have been positively associated with MIH. Some studies link antibiotic use with MIH.^18,19^ However, again it is not possible to be sure, if childhood illness/fever or the treatment with an antibiotic is the causative factor or if both are involved. The use of amoxicillin during the first year of life has been found to increase the risk of MIH^20^ and fluoride-like defects in the permanent incisors and FPMs. Accidental exposure to high levels of dioxins or polychlorinated biphenyls (PCBs) in early childhood has been found to be associated with demarcated opacity and/or hypoplasia.^21,22^ In a Finnish study, a significant correlation between MIH and the exposure of the children to dioxins via mother’s milk was found.^23^ However, in a more recent study of children born 10 years later exposed to lower levels of dioxins, no correlation was found.^24^ The most toxic dioxin congener, 2, 3, 7, 8-tetrachlorodibenzo-para-dioxin (TCDD) arrests degradation and/or removal of enamel matrix proteins in developing molars of rat pups exposed via their dams’ milk, this apparently leads to disturbances in mineralization. Fluoride is thought to affect enamel crystal formation mainly during the maturation stage inducing defects described as diffuse opacities. In three studies, the association between fluoride supplementation and MIH was studied.^23,25,26^ No significant association was found.

## PREVALENCE

Majority of studies report the frequency of MIH in a specific group rather than as prevalence. In nearly half of the studies investigation for MIH was the main purpose. However, it could also be a part of a general dental health survey or combined with an ordinary dental examination. The sample sizes have varied considerably. Descriptions of the study groups were generally sparse or missing. Almost half of the study groups were mixed age groups, three of them^26^ reported prevalence figures for each age group separately which showed a considerable variation between the groups (Table 3).

Traditionally, there have been more studies from Northern Europe and MIH has appeared to be more common in those countries. However, lately studies have been published from other parts of the world. A very recent study from Brazil^46^ showed a prevalence of 40.2% and a study from Kenya^39^ 13.7%.

## TREATMENT APPROACHES

MIH’s clinical management is challenging due to:

1. The sensitivity and rapid development of dental caries in affected PFMs
2. The limited cooperation of a young child
3. Difficulty in achieving anesthesia
4. The repeated marginal breakdown of restorations.

The available treatment modalities for teeth with MIH are extensive, ranging from prevention, restoration, to extraction. A very useful six-step management approach for MIH has been proposed by William et al^32^ (Table 4).

The decision on which treatment should be used is complex and is dependent upon on a number of factors. The commonly identified factors are the severity of the condition, the patient’s dental age and the child/parent’s social background and expectation. A diagrammatic summary of treatment modalities for treating hypo- mineralized first permanent molar is given in Table 5.

## RESTORING HYPOMINERALIZED PERMANENT INCISORS

Labial localization and enamel discoloration often pose an esthetic concern for any child with MIH incisors. Contemporary minimally invasive techniques offer numerous possibilities for treating these lesions. Yellow or brownish- yellow defects are of full thickness and may occasionally respond to bleaching with carbamide peroxide^47^ while those that are creamy-yellow or whitish-creamy are less porous and variable in depth^48^ and can be treated by microabrasion with 18% hydrochloric acid or 37.5% phosphoric acid and abrasive paste.^49^ More pronounced enamel defects might be dealt with by combining the two methods. Conservative approach should be used as the first line of treatment before more invasive treatment such as resin restorations/vesneers or crowns that may create problems, resulting from the large pulp size and immature gingival contours in young incisors.

**Table 3:** **Table 3:** Summary of epidemiological studies for MIH (modified from Jasulaityte et al 2008^27^)

*Country*		*Study*		*Age of children (years)*		*Sample size*		*MIH prevalance*	
Finland		Alaluusua et al^28^ 1996a		12		97		25%	
Finland		Leppaniemi et al^29^ 2001		7-13		488		19.3%	
Sweden		Jalevik et al^30^ 2001		7.6-8.8		516		18.4%	
Denmark		Esmark and Simonsen (1995) in Weerheijm and Mejare 2003^31^		7		5,277		15-25%	
Finland		Alaluusua et al^23^ 1996b		6-7		102		17%	
Sweden		Koch et al^26^		8-13		2,226		3.6-15.4% (depending on year of birth)	
Turkey		Alpoz and Ertugrul (1999) in Weerheijm and Mejare 2003^31^		7-12		250		14.8%	
Slovenia		Kosem et al (2004) in William et al 2006^32^		5-18		3,954		14.4%	
Italy		Calderara et al^33^ 2005		7.3-8.3		227		13.7%	
Bosnia and		Muratbegovic et al^34^ 2007		12		560		12.3%	
Herzegovina								(2.5-32.5%)	
Netherlands		Weerheijm et al^35^ 2001b		11		497		9.7%	
Lithuania		Jasulaityte et al^36^ 2007		6.5-9.5		1,277		9.7%	
(Kaunas)									
Switzerland		Clavadetscher^31^ 1997		7-8		1,671		6.4%	
(Zurich)									
Germany		Preusser^37^ et al 2007		6-12		1,022		5.9%	
(Giessen)									
Greece		Lygidakis et al 2004				2,640		5.7%	
Germany (Dresden)		Dietrich et al^27^ 2003		10-17		378		2.9%	
Wainuiomata		Mahoney EK, Morrison DG^38^ 2009		7-10		522		14.9%	
Kenya		Kemoli AM^39^ 2008		6-8		3,591		13.73%	
Plovdiv, Bulgaria		Kukleva MP, Petrova SG, Kondeva VK, Nihtyanova TI^40^ 2008		7-14		2,960		3.58%	
Hong Kong		Cho SY, Ki Y, Chu V^41^ 2008		12		2635		2.8%	
Istanbul		Kusku OO, Caglar E, Sandalli N^42^ 2008		7-9		147		14.9%	
Dutch National		Jasulaityte L, Weerheijm KL, Veerkamp JS^27^ 2003		9		422		14.3%	
Epidemiological Survey 2003									
Dutch National		Jasulaityte L, Weerheijm KL, Veerkamp JS^27^ 2003		11				9.7%	
Epidemiological									
Survey 1999									
Greece		Lygidakiset al^43^ 2008		5.5-12		3,518		10.2%	
Libya (Benghazi)		Fteita et al^44^ 2006		7-8.9		378		2.9%	
Australia (pediatri dental specialist referral practice)		c Chawla et al 2004^32^ in Williams et al 2006				182 MIH		70% had ≥1 affected FPMs	
UK		Zagdwon et al^45^ 2002		7		307		14.6%	

## CONCLUSION

The prevalence of MIH appears to be increasing and managing affected children is now a common problem for pediatric dentists. Although the etiology is unclear and may, in fact, be multifactorial, children born preterm and those with poor general health or systemic conditions in their first 3 years may develop MIH. The early identification of such children will allow monitoring of their PFMs so that remineralization and preventive measures can be instituted as soon as affected surfaces are accessible. The age of 8 years, when all FPM are usually erupted is the best time for examination. An agreement on procedure and criteria of examination, as wet or dry teeth and minimum size of recorded defect, is needed. Preparing a well-defined method for training and calibration of future examiners is of utmost importance. The complex care involved must address the child’s behavior and anxiety, aiming to provide durable restorations under pain-free conditions. Extensively affected molars may require extracoronal restorations or extraction. Research is needed to clarify etiological factors and improve the durability of restorations in affected teeth. Information on other teeth affected than FPMs and incisors is also desirable.

**Table 4:** **Table 4:** A clinical management approach for permanent first molars affected by MIH

*Steps*		*Recommended procedures*	
Risk identification		Assess medical history for putative etiological factors	
Early diagnosis		Examine at risk molars on radiograph if possible Monitor these teeth during eruption	
Remineralization and desensitization		Apply localized topical fluorides	
Prevention of dental caries and PEB		Institute through oral hygiene home care program Reduce cariogenicity and erosivity of diet Place pit and fissure sealants	
Restorations and extractions		Place intracoronal (resin composite) bonded with self-etching primer adhesive or extracoronal restorations (stainless steel crowns). Consider orthodontic outcomes postextraction	
Maintenance		Monitor margins of restorations for PEB Consider full coronal coverage restorations in the long-term	

**Table 5:** **Table 5:** Summary of treatment modalities for treating hypomineralized first permanent molar

*Restoring hypomineralized first permanent molars*			
Preventive		Topical fluoride application Desensitizing toothpaste Apply a CPP-ACP topical creme daily using a cotton bud Glass ionomer cement (GIC) sealants can provide caries protection and reduce surface permeability
Direct restoration		Cavity margin placement – All defective enamel is removed – Only the very porous enamel is removed, until good resistance of the bur to enamel is felt GIC restorations – Conventional GIC, resin modified GICs (RMGIC) – Adhesive capability to both enamel and dentine – Long term fluoride release – Poorer mechanical properties ‒ Not recommended to be used in stress bearing areas ‒ Be used as an intermediate restoration Composite resin restorations – Longer-term stability compared with other restorative materials – The polyacid modified resin composites ‒ Have good handling characteristics ‒ Release and take up fluoride; and ‒ Have tensile and flexural strength properties superior to GIC and RMGIC, but inferior to that of resin composite ‒ Use of PMRCs in permanent teeth is restricted to nonstress-bearing areas
Full coverage restoration		When PFMs have moderate to severe PEB, preformed stainless steel crowns (SSCs) are the treatment of choice^47^ – Prevent further tooth deterioration – Control tooth sensitivity – Establish correct interproximal contacts and proper occlusal relationships – Are not as technique sensitive or costly as cast restorations – Require little time to prepare and insert – If not adapted properly may produce an open bite, gingivitis or both – Properly placed, SSCs can preserve PFMs with MIH until cast restorations are feasible Partial and full coverage indirect adhesive or cast crown and onlays – Compared to SSCs, cast restorations ‒ Require minimal tooth reduction ‒ Minimize pulpal trauma ‒ Protect tooth structure ‒ Provide high strength for cuspal overlays ‒ Control sensitivity ‒ Maintain periodontal health due to their supragingival margins
Extraction and orthodontic consideration		Timely extraction is a feasible treatment option in cases of: – Severe hypomineralization – Severe sensitivity or pain – Large multi surface lesions – Difficulty of restoration – Inability to achieve local anesthesia – Behavior management problems preventing restorative treatment – Apical pathosis – Orthodontic space requirements, where FPM are heavily restored in the presence of healthy premolars – Crowding distally in the arch and third permanent molars reasonably positioned – Financial considerations precluding other forms of treatment If the orthodontic condition were favorable, the ideal dental age for extracting the defective FPM would be 8.5 to 9 years of age
